# Growth Performance, Survival, Blood Chemistry, and Immune Gene Expression of Channel Catfish (*Ictalurus punctatus*) Fed Probiotic-Supplemented Diets

**DOI:** 10.3390/vetsci9120701

**Published:** 2022-12-16

**Authors:** Khanh Q. Nguyen, Timothy J. Bruce, Oluwafunmilola E. Afe, Mark R. Liles, Benjamin H. Beck, Donald Allen Davis

**Affiliations:** 1 School of Fisheries, Aquaculture, and Aquatic Sciences, Auburn University, Auburn, AL 36849, USA; 2 Department of Fisheries and Aquaculture Technology, Federal University of Technology Akure, Akure 340110, Nigeria; 3 Department of Biological Sciences, Auburn University, Auburn, AL 36849, USA; 4 USDA-ARS Aquatic Animal Health Research Unit, Auburn, AL 36830, USA

**Keywords:** *Bacillus* spp., feed enhancement, aquaculture, feed-based probiotics, nutritional health

## Abstract

**Simple Summary:**

Two trials were performed, utilizing channel catfish fed probiotic-supplemented diets in flow-through systems under natural rearing conditions. There were no significant improvement in growth performance, survival, hematocrit, and blood chemistry parameters in channel catfish fed *Bacillus velezensis*-amended and *Bacillus subtilis*-amended diets. In the first trial, immune gene expression indicated a significant down in *B. velezensis* AP193-fed fish for *il1β*, *tnf-α*, and *tlr9* expression within splenic tissue, compared to that of the basal and *B. subtilis* diets. In the second trial, no substantial up-or down-regulation of immune-related genes was observed in *B. subtilis*-amended feed at different inclusion levels.

**Abstract:**

The channel catfish (*Ictalurus punctatus*) farming industry is the largest and one of the oldest aquaculture industries in the United States. Despite being an established industry, production issues stemming from disease outbreaks remain problematic for producers. Supplementing fish diets with probiotics to enhance the immune system and growth potential is one approach to mitigating disease. Although considerable laboratory data demonstrate efficacy, these results do not always translate to natural modes of disease transmission. Hence, the present work was conducted in the laboratory but incorporated flow-through water from large catfish pond production systems, allowing for natural exposure to pathogens. Two feeding trials were conducted in an 18-tank aquaria system housing two different sizes, 34.8 ± 12.5 g and 0.36 ± 0.03 g, of channel catfish. Channel catfish in the first trial were fed three experimental diets over six weeks. Commercial diets were top-coated with two selected spore-forming *Bacillus* spp. probiotics, *Bacillus velezensis* AP193 (1 × 10^6^ CFU g^−1^) and BiOWiSH (3.6 × 10^4^ CFU g^−1^), or a basal diet that contained no dietary additive. In the second eight-week trial, diets were top-coated with BiOWiSH at three concentrations (1.8, 3.6, and 7.3 × 10^4^ CFU g^−1^), along with one basal diet (no probiotic). At the completion of these studies, growth performance, survival, hematocrit, blood chemistry, and immune expression of interleukin 1β (*il1β*), tumor necrosis factor-alpha (*tnf-α*), interleukin-8 (*il8*), transforming-growth factor β1 (*tgf-β1*), and toll-like receptor 9 (*tlr9*) were evaluated using qPCR. Trial results revealed no differences (*p* > 0.05) among treatments concerning growth, survival, or hematological parameters. For immune gene expression, interesting trends were discerned, with substantial downregulation observed in *B. velezensis* AP193-fed fish for *il1β*, *tnf-α*, and *tlr9* expression within splenic tissue, compared to that of the basal and BiOWiSH diets (*p <* 0.05). However, the results were not statistically significant for anterior kidney tissue in the first trial. In the second trial, varied levels of probiotic inclusion revealed no significant impact of BiOWiSH’s products on the expression of *il1β*, *tnf-α*, *il8*, and *tgf-β1* in both spleen and kidney tissue at any rate of probiotic inclusion (*p* > 0.05). Based on these findings, more research on utilizing probiotics in flow-through systems with natural infection conditions is crucial to ensure consistency from a controlled laboratory scale to real-world practices.

## 1. Introduction

Concentrated in the southern part of the US, the channel catfish (*Ictalurus punctatus*) industry is well-established in the domestic aquaculture sector. This industry has a long developmental history and socioeconomics, coupled with vital research and extension programs. Modernized practices, such as applying intensive aeration in production, adopting split-pond systems, and breeding programs, has propelled channel catfish farming to become one of the largest and oldest aquaculture sectors in the US [1]. Economically, among other farmed freshwater fish, the channel catfish industry alone contributed about $352 million U.S. dollars in sales annually in 2021 [2]. This is a sizable contribution, given that the majority of the industry’s revenue comes from four major states: Mississippi, Alabama, Arkansas, and Texas. Despite their tolerance of poor water quality and resilience to several infectious agents, as well as well-established business models, expanding the production of channel catfish and other farmed aquatic animals for human consumption is fraught with challenges connected to biotic and abiotic factors, notably disease outbreaks. In fact, pathogenic infections, such as bacterial, fungal, and parasitic diseases, have caused very high mortality in channel catfish aquaculture, including motile *Aeromonas* septicemia (MAS), enteric septicemia of catfish (ESC), and columnaris disease [3,4,5,6,7]. Despite antibiotics’ considerable efficacy in preventing and managing both infectious and non-infectious diseases, concerns about antibiotic resistance, costs, and residue accumulation may outweigh the advantages of antibiotics in the long run, making them less sustainable [8,9,10,11]. Numerous strategies have been evaluated to limit antibiotic usage, some of which use probiotics and herbal extracts or innovative methods, such as vaccination or interference of quorum sensing via probiotics [12,13,14,15,16,17,18].

Among the possible antibiotic alternatives, feed additives, particularly probiotics, are economical, relatively simple to administer, and scalable, depending on the size of the production operation. Probiotics have shown promise in preventing and managing pathogenic agents, contributing to better water quality, promoting animal health, and accelerating growth [19,20,21]. Probiotic amendments have demonstrated usefulness in various farming systems for many species ranging from teleost fish to crustaceans in improving innate immunity, competing for limiting factors, and decreasing the population of pathogenic bacteria to a tolerable density that limits illness risk, particularly by generating a healthy gastrointestinal microbiota that promotes fish growth [22,23,24,25]. Various bacterial candidates have been identified and isolated for aquaculture application, of which *Bacillus* spp. is most dominant within the commercially-available products, especially for dietary inclusion, along with water-amended products using nitrifying bacteria [26]. *Bacillus* spp. isolates from soybean or other plant rhizospheres may be well-suited as additions to soy-based fish feed, as previous studies had shown they could colonize the intestinal tracts of several aquatic species [27,28]. Although there are numerous accounts of significant proof of efficacy in the laboratory, these findings are not always transferable to production-scale settings or relevant to natural routes of disease transmission. Interestingly, studies have shown that the efficiency of probiotics is significantly impacted by environmental conditions, which can either impair or negate the benefits of a microorganism-enriched diet [29,30,31].

In order to assess the effects of probiotics on channel catfish growth performance, survival, blood chemistry, and immune gene expression, two feeding trials using catfish production pond water were conducted. The flow-through water from the effluent of a large catfish pond production system was utilized to more closely approximate traditional pond-rearing conditions.

## 2. Materials and Methods

### 2.1. Diet Preparation

The basal diet (BD) was formulated to 32% protein and 6.5% lipid (Table 1). The BD was made at Aquatic Animal Nutrition Laboratory at the School of Fisheries, Aquaculture, and Aquatic Sciences, Auburn University (Auburn, AL, USA), utilizing standard fish feed procedures. The pre-ground dry ingredients and oil were weighed and then incorporated for 15 min in a food mixer (Hobart Corporation, Troy, OH, USA). The mixture was then mixed with hot water to get a pellet-ready consistency. Diets were pressure-pelleted with a 3-mm die on a meat grinder.

Afterward, the moist pellets were put in a forced air oven (<45 °C) overnight to achieve less than 10% moisture content. Dry pellets were crumbled, packed in bags, and kept in a freezer (−20 °C) until needed. The proximate composition and amino acid (AA) profile of all diets were determined at the University of Missouri Agriculture Experiment Station Chemical Laboratories (Columbia, MO, USA; Table 1).

### 2.2. Probiotics and Test Diets

Spores of the two probiotic strains were sprayed onto the BD as a top coat. For *Bacillus velezensis* AP193, 0.025 g kg^−1^ of a lyophilized spore stock determined to be 4 × 10^10^ colony forming units (CFU) per g was suspended in 10 mL of distilled, deionized water and sprayed onto feed for a final concentration of 1 × 10^6^ CFU g^−1^ (Table 2, B-AP). For BiOWiSH, the *Bacillus subtilis* FeedBuilder Syn3 spore stock was 7.2 × 10^7^ CFU g^−1^ (BiOWiSH Technologies Inc., Cincinnati, OH, USA), which was suspended in distilled, deionized water, according to manufacturer’s specifications, and sprayed onto feed for a final concentration in the first experiment of 3.6 × 10^4^ CFU g^−1^ (Table 2, B-BW). In the second experiment, the final concentrations of the *B. subtilis* FeedBuilder Syn3 on feed were 0 (basal diet), 1.8, 3.6, and 7.2 × 10^4^ CFU g^−1^ (Table 2; B-BW-L, B-BW, B-BW-H).

### 2.3. Water Quality

Dissolved oxygen was maintained near saturation using air stones in each culture tank, and the sump tank using a standard airline connected to a regenerative blower. During the trial, dissolved oxygen (DO), temperature, and salinity were monitored twice daily using a YSI 55 multi-parameter instrument (YSI, Yellow Springs, OH, USA). Total ammonia N (TAN) and nitrite-N were measured twice per week using YSI 9300 photometer (YSI, Yellow Springs, OH, USA). The pH of the water was measured twice weekly during the experimental period using the EcoSense pH10A (YSI, Yellow Springs, OH, USA).

### 2.4. Experiment A: Probiotic Assessment

The first 6-week experiment took place in a biosecure wet lab at E. W. Shell Fisheries Center of Auburn University, Auburn, Alabama, from March to May 2021. Ten juvenile channel catfish (34.8 ± 12.5 g) were randomly stocked into twelve aquaria (75 L) in a flow-through system utilizing natural water sourced from channel catfish production ponds. Catfish were hand-fed twice daily at ~4% body weight, and the ration was adjusted every 2 weeks. The probiotic spores were top coated on fish feed with a final concentration of *B. velezensis* AP193 at 1 × 10^6^ CFU g^−1^ (B-AP), and for BiOWiSH, the final concentration was 7.2 × 10^4^ CFU g^−1^ (B-BW). The control or basal diet without probiotic amendment was coated with distilled water. The diets were then air-dried for at least 12 h, stored at 4 °C, and used within 3 days of mixing. Each experimental diet was administered to 6 replicate tanks for the study duration.

The study tanks received water from channel catfish production ponds with a mean water flow rate of 1 L min^−1^. During the trial, the water quality was within range for normal growth (6.78 ± 0.13 mg L^−1^ dissolved oxygen, 0.36 ± 0.11 mg L^−1^ total ammonium nitrogen (TAN), 0.03 ± 0.02 mg L^−1^ nitrite, 0.14 ± 0.03 g L^−1^ salinity, and pH 8.36 ± 0.71), except for temperature (20.01 ± 0.33 °C) [32].

At the end of the feeding trial, fish were bulk weighed, and three fish were randomly collected from each tank, anesthetized with buffered tricaine methanesulfonate (MS-222), and bled from the caudal vein with a 1 mL syringe, and then fish were euthanized, and the spleen and anterior kidney tissues were collected.

Blood samples were collected in a 1.5 mL microcentrifuge tube without anticoagulant for biochemistry analysis. For hematocrit analysis, blood was collected in heparinized soda-lime glass micro-hematocrit capillary tubes (DWK Life Sciences LLC, Milville, NJ, USA) that were wax-sealed (Paul Marienfeld GmbH & Co. KG, Lauda-Königshofen, Germany). Spleen and kidney tissues were collected and preserved in DNA/RNA Shield (Zymo Research, Irvine, CA, USA) within 1.5 mL microcentrifuge tubes for gene expression analysis. All growth metrics were calculated as follows:

$$Final\;weight\;\left(FW,\;g\right)=\frac{Total\;biomass\;\left(g\right)}{Number\;of\;fish\;at\;termination\;\left(g\right)}$$

$$Percent\;weight\;gain\;\left(WG;\%\right)=\frac{Final\;weight\;\left(g\right)-Initial\;weight\;\left(g\right)}{Initial\;weight\;\left(g\right)}\;\times \;100$$

$$Survival\;rate\;\left(SR;\%\right)=\frac{1-Total\;recorded\;mortalities}{Number\;of\;fish\;at\;the\;start\;of\;the\;study}\;\times \;100\;$$

Thermal−unit growth coefficient (TGC)=Final weight13−Initial weight13Temperature (°C) × Days × 1000

### 2.5. Experiment B: Growth and Flow-Through with Juvenile Channel Catfish

The second 8-week growth trial was conducted from August to October 2021, using fingerling channel catfish (0.36 ± 0.03 g) randomly stocked into 18, 105 L fiberglass tanks in a flow-through system with the stocking density at 40 fish tank^−1^ using a natural water source from channel catfish production ponds. Fish were hand-fed twice daily at ~4% body weight, and the ration was adjusted every 2 weeks. There were four experimental diets, basal diet, and three inclusion levels of BiOWiSH FeedBuilder Syn3, with a final dosage on feed of 1.8 × 10^4^, 3.6 × 10^4^, and 7.2 × 10^4^ CFU g^−1^ top-coated on feed. The inclusions represented 50, 100, and 200% of the recommended dose (B-BW-L, B-BW, and B-BW-H, respectively). The diets were left air-dried for at least 12 h, stored at 4 °C, and used within 3 days. The experimental diet was administered to 4 replicate tanks for 0.25 g kg^−1^ and 0.5 g kg^−1^ inclusion levels, while 0 g kg^−1^ and 1 g kg^−1^ had five replicate tanks for the study duration.

The study tanks received water from channel catfish production ponds, with the mean water flow rate at 1 L min^−1^. Similar to the first trial, the water quality was within range for the normal growth for channel catfish (6.48 ± 0.04 mg L^−1^ dissolved oxygen, 0.14 ± 0.03 mg L^−1^ total ammonia nitrogen, 0.03 ± 0.01 mg L^−1^ nitrite, 0.19 ± 0.09 g L^−1^ salinity, 8.06 ± 0.09 pH, and temperature (27.51 ± 0.19 °C) [32].

At the end of the feeding trial, fish were bulk-weighed, and three fish were collected, as previously described, for blood, spleen, and kidney samples. All growth parameters were calculated as similar to the first trial, with the addition of:

$$Feed\;conversion\;ratio\;\left(FCR\right)=\frac{Feed\;fed\;for\;the\;entire\;study\;\left(g\right)}{Biomass\;gained\;during\;study\;\left(g\right)}\;\times \;100$$

### 2.6. Hematocrit Analysis

Wax-sealed capillary tubes were spun down using a hematocrit IEC Clinical Centrifuge (International Equipment Co., Needham Heights, MA, USA) in 5 min using the instrument setting. The hematocrit percentage results were then read using a micro-capillary reader (International Equipment Co., Needham Heights, MA, USA).

### 2.7. Serum Biochemistry Analysis

Blood samples were allowed to clot at 4 °C overnight, followed by centrifugation at 15,000× *g* for 5 min to collect serum. Three serum samples from each tank were then pooled into one 100 μL composite sample. The serum biochemical parameters (alkaline phosphatase, alanine transaminase, gamma-glutamyl transferase, bile acids, total bilirubin, albumin, blood urea nitrogen, and cholesterol) were determined by using Abaxis VetScan Mammalian Liver Profile on the Abaxis VetScan VS2 analyzer (Zoetis, Union City, CA, USA).

### 2.8. qPCR Gene Expression Analyses

RNA of spleen and kidney samples were extracted and purified using Quick-RNA Miniprep Kit (ZYMO Research, Irvine, CA, USA). Sample concentration was measured using a NanoDrop One^c^ microvolume spectrophotometer (Thermo Fisher Scientific, Waltham, MA, USA). Extracted RNA samples were then diluted and standardized to 50 ng μL^−1^. All samples were then converted into cDNA using the High-Capacity cDNA Reverse Transcription Kit (Applied Biosystems, Waltham, MA, USA), according to the manufacturer’s instructions. A total of 20 μL was used in the reaction, which included 2 μL of 10× R.T. buffer, 0.8 μL of 25× dNTP Mix, 2 μL of 10× R.T random primers, 1 μL of multiscribe reverse transcriptase, and 4.2 μL of nuclease-free water. The cDNA was synthesized using a MiniAmp Plus thermal cycler (Applied Biosystems, Carlsbad, CA, USA). The thermal program was set at 25 °C for 10 min, 37 °C for 120 min, and 85 °C for 5 min. The RNA with a 25 ng μL^−1^ concentration was diluted to reach the concentration of 2.5 ng μL^−1^. Experiment A utilized four genes: *il1β* (interleukin 1 beta), *tnf-α* (tumor necrosis factor alpha), *tlr9* (toll-like receptor 9), and *tgf-β1* (transforming growth factor beta 1) with a housekeeping gene (*18s* rRNA), while Experiment B used four genes *il1β*, *tnf-α*, *il8*, and *tgf-β1* with two housekeeping genes: *ef1α* (elongation factor 1 alpha) and *actb* (beta-actin) (Table 3). The efficiencies of the primers were determined by performing five serial dilutions, with a dilution ratio of 1:10, to achieve 90% to 110% efficiency for each gene. Totals of 5 μL of Powerup SYBR Green Master Mix (Applied Biosystems, Carlsbad, CA, USA), 0.5 μL of each forward and reverse primer (stock concentration of 100 μM), 2 μL of nuclease-free water, and 2 μL of cDNA sample were used in each 10 μL reaction. Each sample was analyzed in duplicate, along with a negative control (nuclease-free water in place of a cDNA template). QuantStudio 5 Real-time PCR (Applied Biosystems, Carlsbad, CA, USA) was used for all runs, with cycle settings of 50 °C for 2 min, 95 °C for 2 min, followed by 40 cycles of 95 °C for 15 s, 58 °C for 15 s, and 72 °C for 30 s. All relative quantifications were calculated according to the comparative Ct method (2^−ΔΔCt^) [33].

### 2.9. Statistical Analysis

All data were analyzed using R Version 4.2.1 (R Foundation for Statistical Computing, Vienna, Austria). The natural log transformation was performed on two parameters for blood biochemistry (ALP and ALT) and all relative gene expressions to meet the normality requirement [40,41]. Outliers were detected and removed by using Dixon’s test. Residuals were tested for normality using the Shapiro–Wilk test and equivalent variances using Bartlett’s test. Analysis of variance (ANOVA) was used to compare treatment results, and significant outcomes were tested post-hoc using Tukey’s honest significant difference for multiple comparisons. An a priori alpha value of *α* = 0.05 was used for all statistical analyses. The pooled standard error (PSE) was calculated as follows:

$$Pooled\;standard\;error\;\left(PSE\right)=\frac{Root\;mean\;squared\;error}{\sqrt{Average\;number\;of\;replicates}}$$

## 3. Results

### 3.1. Growth Performance

After six weeks, the two diets amended with *B. velezensis* B-AP and B-BW *B. subtilis* probiotics of Experiment A revealed no statistically significant changes (*p* > 0.05) in the final weight, percent weight gain, survival rate, or thermal-unit growth coefficient among treatments (Table 4). Experiment B yielded similar results after eight weeks, with no discernible difference for varying levels of inclusion in the final weight (*p* = 0.122), percent weight gain (*p* = 0.090), survival rate (*p* = 0.715), feed conversion ratio (*p* = 0.228), and thermal-unit growth coefficient (*p* = 0.123) (Table 5).

### 3.2. Hematological and Blood Serum Parameters

Similar patterns were identified for hematocrit and serum biochemistry parameters in Experiment A, despite various tendencies for bile acids, in which the basal diet presented the lowest level and comparatively lower hematocrit quantity for the *B. velezensis*-supplemented diet (B-AP). However, there was no significant difference discovered (*p* = 0.462; Table 6). Experiment B revealed a similar tendency for bile acids, which decreased with the B-BW treatment. In contrast, hematocrit fluctuated at different levels of probiotics inclusion, but there was no statistically significant difference (*p* = 0.570) (Table 7).

### 3.3. Gene Expression

**Figure 1:**
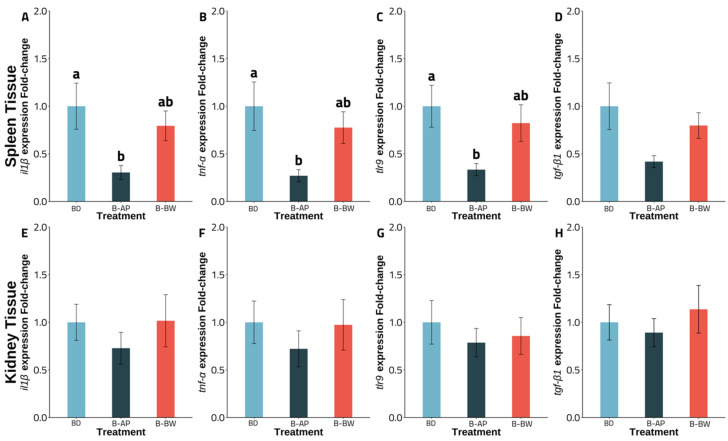
Gene expression of channel catfish from experiment A fed basal (BD; *n* = 5), *B. velezensis* AP193 (B-AP; *n* = 4), or BiOWiSH FeedBuilder Syn3 (B-BW; *n* = 6) probiotic-amended diet during a 6-week period with the initial weight of 34.80 ± 12.53 g. Different letters indicate a significant treatment difference (*p* < 0.05). Bar graphs presented as mean and error bars, as standard error of the mean. BD = basal diet, B-AP = *B. velenzesis* included, B-BW = BiOWiSH FeedBuilder Syn3 included. Figure (**A**–**D**): Expression of *il1β* (**A**), *tnf-α* (**B**), *tlr9* (**C**), and *tgf-β1*(**D**) of spleen tissue. Figure (**E**–**H**): Expression of *il1β* (**E**), *tnf-α* (**F**), *tlr9* (**G**), and *tgf-β1* (**H**) of kidney tissue.

**Figure 2:**
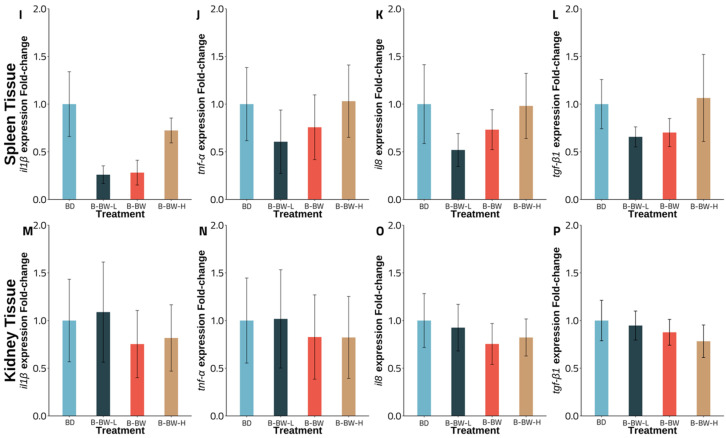
Gene expression of channel catfish from experiment B fed BiOWiSH probiotics diet, with three different probiotic inclusion levels (B-BW-L; *n* = 4, B-BW; *n* = 4, B-BW-H; *n* = 5), and a basal diet (BD; *n* = 5) during an 8-week period with the initial weight of 0.36 ± 0.03 g. Different letters indicate a significant difference (*p* < 0.05). Bar graphs are presented as mean and error bars, as the standard error of the mean. BD = basal diet; BiOWiSH FeedBuilder Syn3 with low 1.8 × 10^4^ CFU g^−1^ (BW-L), recommended 3.6 × 10^4^ CFU g^−1^ (B-BW), and high 7.2 × 10^4^ CFU g^−1^ (B-BW-H) concentrations of *B. subtilis*. Figure (**I**–**L**): Expression of *il1β* (**I**), *tnf-α* (**J**), *il8* (**K**), and *tgf-β1* (**L**) of spleen tissue. Figure (**M**–**P**): Expression of *il1β* (**M**), *tnf-α* (**N**), *il8* (**O**), and *tgf-β1* (**P**) of kidney tissue.

Experiment A gene expression revealed an intriguing pattern for fish fed with a *B. velezensis* AP193-amended diet, with a significant down-regulation observed for the *il1β*, *tnf-α*, and *tlr9* transcripts from spleen tissue, compared to that of the basal diet (*p* < 0.05). No significant changes were observed for *tgf-β1* in spleen tissue in the control, compared to the *B. velezensis* AP193-amended diet. At the same time, there was no significant difference, in comparison to the B-BW diet (*p* > 0.05) (Figure 1). Although a similar pattern was found in kidney tissue, there was no statistical significance in the down-regulation of the immune-related genes between the two probiotics and the basal diets (*p* > 0.05) (Figure 1). In Experiment B, varied levels of probiotics inclusion revealed no significant impact of BiOWiSH-amended feed on the expression of *il1β*, *tnf-α*, *il8*, and *tgf-β1* in either the spleen or kidney tissue with the B-BW-L and B-BW treatments (Figure 2). Furthermore, although having twice the suggested inclusion level, B-BW-H had no significant effects on immune gene expression in either organ (*p* > 0.05) (Figure 2).

## 4. Discussion

Probiotics have been promoted and employed as a solution to accelerate growth and improve immune responses, given that antimicrobial resistance has resulted in a limited number of drugs that can be used to battle pathogen infections, as well as the cost and scale concerns of immunization [10,17,42,43,44,45]. Furthermore, prior research found that, in addition to promoting development, *B. velezensis* AP193 was observed to reduce eutrophication in channel catfish ponds, showing the possibility for employing this probiotic to improve culture systems water quality [30]. On the other hand, Ran et al. (2012) reported that bacterial retention was decreased in the flow-through system, resulting in a loss of *B. velezensis* AP193’s protective potential against pathogenic bacteria [27].

The current study found no significant difference in the growth performance of fish offered diets with *B. velezensis* AP193 or BiOWiSH probiotics and the basal diet, in terms of final weight (g), weight gain (%), survival rate (%), feed conversion ratio, and thermal-unit growth coefficient. Furthermore, despite amending feed with 2× the recommended level (B-BW-H, 7.2 × 10^4^ CFU g^−1^, or 1 g kg^−1^ product inclusion), the second study showed the same outcome, with no significant difference between probiotic-supplemented and basal diets in the flow-through system. This is consistent with prior research by Peterson et al. (2010), in which channel catfish provided *Lactobacillus* spp. and *Bacillus* spp. displayed no discernible change in growth and survival rates [46]. Furthermore, Merrifield et al. (2010) found no significant differences in the final weight, weight gain, or survival rate of rainbow trout (*Oncorhynchus mykiss*) fed *Bacillus licheniformis* and *Bacillus subtilis* [47]. Concerning invertebrates, Hai et al. (2009) found no statistically significant difference in the survival rate or feed conversion ratio between the direct and indirect incorporation of probiotics in diets for western king prawns (*Penaeus latisulcatus*) [48]. Concerning the results of trial 1, it should be noted that the number of viable probiotic spores added to these diets were different between the two probiotics, and the concentrations used were lower than in another study, in which significant increases in channel catfish growth were reported [30].

Hematological and blood serum parameters have been used to assess the physiological conditions of the animals; evaluating the related parameters may bring a better understanding of the impacts of probiotic-amended diets on the health of the vital organs and, ultimately, the cultured animals [49,50]. Regarding hematocrit and blood serum chemistry, Panigrahi et al. (2010) reported no difference in hematocrit values in rainbow trout between treatments for the first 20 days [51]. A significant difference was observed at 30 days between the control and freeze-dried probiotic diets, but no difference between the control and heat-killed probiotic-fed group. Similarly, it has been previously reported that channel catfish fed yeast diets exhibited no changes in white blood cells, red blood cells, hematocrit, or hemoglobin levels at the end of the study [52]. On the other hand, despite the lack of meaningful evidence on the effects of probiotics on total cholesterol and alkaline phosphatase during the first ten days of the trial, Panigrahi et al. (2010) again reported obvious observable patterns after 20 days [51]. Thus, feeding times for probiotic-supplemented diets may play a role in their ability to exert influences on fish health and enzyme activity. Nonetheless, after eight weeks, Asian sea bass (*Lates calcarifer*) fed a probiotic diet exhibited considerably greater levels of all hematological indices and hepatic enzyme activity, including hematocrit, red blood cell, white blood cell, hemoglobin, alanine aminotransferase, and alkaline phosphatase [53]. Nevertheless, Aly et al. (2008) found comparable results to Reda and Selim (2015) in which hematological indicators showed a substantial difference between the probiotics and control groups, but no difference between the probiotic-fed treatments [54,55]. Both trials of this current investigation revealed no statistically significant differences between the probiotics and control groups, indicating that, within this experimental design, the probiotics had little to no impact on blood serum activity and hematological markers, even at higher inclusion levels (Table 7).

Although the regulation remained similar for varying degrees of BiOWiSH inclusion, significant downregulations in spleen tissue for the *il1β* (*p = 0.029*), *tnf-α* (*p = 0.024*), and *tlr9* (*p = 0.040*) genes were observed for the *B. velezensis* AP193-amended diet group, while kidney tissue expression remained the same for all four genes (*p > 0.05*). Given that the investigated genes are proinflammatory cytokines and cell receptor genes, particularly *il1β*, *tnf-α*, *tlr9*, *tgf-β1*, and *il8*, the up- or down-regulation of any of these genes suggests the modulation of an immune response [37,56]. IL-1β, a product of blood monocytes and tissue macrophages, is essential for leukocyte movement, lymphocyte activation, and other bactericidal functions [57]. The current experiment had a similar effect to Picchietti et al. (2009), with fish in the probiotic-fed group showing a substantial reduction in inflammatory markers, compared to the control groups [58]. Furthermore, the research showed that cortisol, an immunosuppressive factor, decreased dramatically in the probiotic-altered group. On the other hand, TGF-β1, another member of the cytokine family, is involved in signaling cell formation, proliferation, and migration in leukocytes and is closely associated with *il1β*. Hence, a decrease in *il1β* was found alongside a decrease in *tgf-β1* [57,58,59]. In contrast with this study, Mohammadian et al. (2021) indicated a significant change in the regulation of *tgf-β1* for head kidney tissue in shabout (*Tor grypus*) fed a probiotic diet [60]. Tumor necrosis factor α, TNF-α, an inflammatory mediator, stimulates phagocytosis and macrophage activity to attack the intruder [61]. Concerning no significant change in *tnf-α* in the kidney tissue of the *B. velezensis* AP193 group in this study, a contrast finding in olive flounder (*Paralichthys olivaceus*) fed *Lactobacillus* signified a noticeable upregulation of *tnf-α* in the anterior kidney, compared to the control group [62]. Furthermore, the gene expression of olive flounder fed *Lactococcus lactis* subsp. showed a noticeable upregulation in spleen tissue, while no significant impact was found for kidney tissue [63]. IL8, a protein produced by macrophages and monocytes, leads to the migration of neutrophils to the inflammation site [64]. Despite the promotion in the regulation of *il8* in the head kidney, the study of Mohammadian et al. (2021) and Rodríguez et al. (2009) contrasted with the outcome of this trial and that of Lu et al. (2020), for which no up- or down-regulation could be observed even with different level of BiOWiSH and MOS, respectively [60,65,66]. Cell receptor TLR9, on the other hand, allows the innate immune system to produce proinflammatory cytokines and interferon [67]. The findings of this study indicated that *B. velezensis* AP193 contributed to a decrease in *tlr9* expression in channel catfish in the spleen, but not in anterior kidney tissue, but this was different with the findings of Liu et al. (2020) in the kidney of golden pompano (*Trachinotus ovatus*), which expressed upregulation in the probiotics added diet group [68]. Furthermore, Wang et al. (2020) reported substantial upregulation of *tlr9* in yellow catfish (*Pelteobagrus fulvidraco*) after a challenge with *Flavobacterium columnare* [69]. In the current study, we found that *B. velezensis* AP193 had no substantial deleterious influence on the integrity of essential organs, such as the spleen.

## 5. Conclusions

Probiotic administration has been shown to boost growth rate and the immunological response in cultured aquatic animals. Not all strains that demonstrate good benefits in vitro would function similarly on a larger production scale. Additionally, different species may react differently to probiotic treatments, especially when the required rearing conditions are quite different. Our current study findings did not discern growth and survival differences with probiotic treatment additions, but did reveal changes to immune gene expression in the splenic tissue of fish fed the diet containing *B. velezensis* AP193. Various diets with diverse component matrices may boost or hinder the development of bacteria; studies on inoculating beneficial microorganisms with regularly used aquaculture ingredients should be conducted, and both ingredients used in the current study can be further examined for optimized dosing and life-stage specific administration for channel catfish production.

## Figures and Tables

**Table 1:** Formulation and proximate composition of basal diet (BD) used in the feeding trials (% as is).

**Ingredients ^1^**	**BD**	**Amino Acids ^1^**	**BD**
Poultry meal ^a^	6.00	Alanine	1.60
Soybean meal ^b^	55.50	Arginine	2.34
Menhaden fish oil ^c^	3.59	Aspartic Acid	3.53
Corn Starch ^d^	3.46	Cysteine	0.49
Corn ^e^	28.00	Glutamic Acid	5.77
Mineral premix ^f^	0.50	Glycine	1.64
Vitamin premix ^g^	0.80	Histidine	0.86
Choline chloride ^h^	0.20	Hydroxylysine	0.08
Rovimix Stay-C ^i^	0.10	Hydroxyproline	0.25
CaP-dibasic ^j^	1.85	Isoleucine	1.62
		Lanthionine	0.04
		Leucine	2.63
		Lysine	2.08
		Methionine	0.52
		Ornithine	0.04
		Phenylalanine	1.68
		Proline	1.76
		Serine	1.13
		Taurine	0.17
		Threonine	1.17
		Tryptophan	0.42
		Tyrosine	1.16
		Valine	1.76
**Proximate composition ^1^ (g/100g as is)**			
Crude protein	33.7		
Moisture	6.57		
Crude Fat	4.85		
Crude Fiber	4.24		
Ash	6.63		

^a^ Tyson Foods, Inc., Springdale, AR, USA. ^b^ De-hulled Solvent Extracted Soybean Meal, Bunge Limited, Decatur, AL, USA. ^c^ Omega Protein Inc., Houston, TX, USA. ^d^ MP Biomedicals Inc., Solon, OH, USA. ^e^ Faithway Feed Co., Gunterville, AL, USA. ^f^ Trace mineral premix (g/100g premix): Cobalt chloride, 0.004; Cupric sulfate pentahydrate, 0.250; Ferrous sulfate, 4.000; Magnesium sulfate anhydrous, 13.862; Manganese sulfate monohydrate, 0.650; Potassium iodide, 0.067; Sodium selenite, 0.010; Zinc sulfate heptahydrate, 13.193; Alpha-cellulose, 67.964. ^g^ Vitamin premix (g/kg premix): Thiamin HCl, 0.438; Riboflavin, 0.632; Pyridoxine HCl, 0.908; Ca-Pantothenate, 1.724; Nicotinic acid, 4.583; Biotin, 0.211; folic acid, 0.549; Cyanocobalamin, 0.001; Inositol, 21.053; Vitamin A acetate, 0.677; Vitamin D3, 0.116; Menadione, 0.889; dL-alpha-tocoperol acetate, 12.632; Alpha-cellulose, 955.589. ^h^ VWR Amresco, Suwanee, GA, USA. ^i^ Stay-C^®^ (L-ascorbyl-2-polyphosphate 35% Active C), Roche Vitamins Inc., Parsippany, NJ, USA. ^j^ VWR Amresco, Suwanee, GA, USA ^l^ Analysis conducted by University of Missouri Agricultural Experimental Station Chemical Laboratories (Columbia, MO, USA) (Results are expressed on g/100 g of feed as is, unless otherwise indicated).

**Table 2:** Experimental diets abbreviations of probiotic types, inclusion levels, and concentrations fed to channel catfish.

Diet Abbreviations	Probiotic	Dietary Inclusion Level (g kg^−1^)	Product Stock Concentration (CFU g^−1^)	Product Concentration on Feed (CFU g^−1^)
**Experiment A**				
BD				
B-AP	*B. velezensis*	0.025	4.0 × 10^10^	1.0 × 10^6^
B-BW	*B. subtilis*	0.5	3.6 × 10^7^	3.6 × 10^4^
**Experiment B**				
BD				
B-BW-L	*B. subtilis*	0.25	1.8 × 10^7^	1.8 × 10^4^
B-BW	*B. subtilis*	0.5	3.6 × 10^7^	3.6 × 10^4^
B-BW-H	*B. subtilis*	1.0	7.2 × 10^7^	7.2 × 10^4^

**Table 3:** Primers used for real time qPCR analysis.

Gene	Accession Number	Amplification Size (pb)	Forward Primer (5′ to 3′)	Reverse Primer (5′ to 3′)	Reference
**Cell receptor**					
***tlr9***	HQ677720	110	GGAGGAACGGGACTGGATAC	AAGCACAGCCACCCTGATTA	[34]
**Cytokines**					
***il1β***	NM001200220.1	180	GTGTAAGCAGCAATCCAGTCA	CAAGCACAGAACAGTCAGGTAT	[35]
***tnf-α***	NM_001200172.1	277	GGCCTCTACTTCGTCTAC	GCAGCAGCTTCTCGTCCAT	[35]
***tgf-β1***	JT417317 ENA	167	GAAACATCCCAGCACCTCCA	GCCAAGCAAACAACGGCTAA	[34]
***il8***	AY145142	264	CAATACTTTGTGAATTTCTGC	TGTCCTTGGTTTCCTTCTGG	[36]
**Reference gene**					
***18S***	AF021880		GAGAAACGGCTACCACATCC	GATACGCTCATTCCGATTACAG	[37]
***ef1α***		118	GTTGAAATGGTTCCTGGCAA	TCAACACTCTTGATGACACCAAC	[38]
***actb***		139	CCGTGACCTGACTGAATACC	GCCCATCTCCTGCTCAAAG	[39]

**Table 4:** Growth performance of channel catfish cultured in flow-through system for 6 weeks fed different probiotics, stocked at 10 fish/tank with an initial weight at 34.80 ± 12.53 g (Mean ± SEM). BD = basal diet, B-AP = *B. velenzesis* included, B-BW = BiOWiSH FeedBuilder Syn3 included.

Parameters	BD	B-AP	B-BW	PSE ^a^	*p*-Value
Final average weight (g)	52.46	50.19	50.45	2.179	0.727
Percent weight gain (%)	47.60	42.76	42.84	6.669	0.843
Survival rate (%)	95.00	100.00	98.33	1.610	0.116
Thermal-unit growth coefficient	3.07	2.71	2.76	0.414	0.808

^a^ PSE = Pooled standard error.

**Table 5:** Growth performance of channel catfish cultured in flow-through system for 8 weeks fed different inclusion levels of BiOWiSH, stocked at 40 fish tank^−1^ with an initial weight at 0.36 ± 0.03 g (Mean ± SEM). BD = basal diet; BiOWiSH FeedBuilder Syn3 with low 1.8 × 10^4^ CFU g^−1^ (BW-L), recommended 3.6 × 10^4^ CFU g^−1^ (B-BW), and high 7.2 × 10^4^ CFU g^−1^ (B-BW-H) concentrations of *B. subtilis*.

Parameters	BD ^a^	B-BW-L ^b^	B-BW ^b^	B-BW-H ^a^	PSE ^c^	*p*-Value
Final average weight (g)	21.78	21.19	18.92	21.17	0.806	0.122
Percent weight gain (%)	5832.31	6241.15	5075.88	5680.73	280.9	0.090
Survival rate (%)	99.00	100.00	98.75	97.00	1.863	0.715
Feed conversion ratio	0.92	0.91	0.99	0.92	0.027	0.228
Thermal-unit growth coefficient	1.57	1.53	1.36	1.53	0.059	0.123

^a^*n* = 5, ^b^ *n* = 4, ^c^ PSE = Pooled Standard Error.

**Table 6:** Hematological parameters of channel catfish cultured in flow-through system for 6 weeks fed different probiotics, stocked at 10 fish tank^−1^ with an initial weight at 34.80 ± 12.53 g (Mean ± SEM). BD = basal diet, B-AP = *B. velenzesis* included, B-BW = BiOWiSH FeedBuilder Syn3 included.

Parameters	BD	B-AP	B-BW	PSE ^a^	*p*-Value
Alkaline phosphatase (U/L)	3.58	3.57	3.60	0.059	0.948
Alanine transaminase (U/L)	3.71	3.65	3.63	0.108	0.861
Gamma-glutamyl transferase (U/L)	3.50	3.17	3.67	0.292	0.484
Bile acids (µmol/L)	7.00	14.67	14.33	4.804	0.462
Total bilirubin (mg/dL)	0.38	0.30	0.32	0.053	0.521
Albumin (g/dL)	1.40	4.23	1.62	1.479	0.347
Blood urea nitrogen (mg/dL)	3.50	4.17	3.83	0.240	0.179
Cholesterol (mg/dL)	224.00	238.50	256.50	12.601	0.222
Hematocrit (%)	26.94	22.33	25.72	3.598	0.652

^a^ PSE = Pooled Standard Error.

**Table 7:** Hematological parameters of channel catfish cultured in flow-through system for 8 weeks fed different inclusion levels of BiOWiSH (0 to 7.2 × 10^7^ CFU g^−1^), stocked at 40 fish tank^−1^ with an initial weight at 0.36 ± 0.03 g (Mean ± SEM). BD = basal diet; BiOWiSH FeedBuilder Syn3 with low 1.8 × 10^4^ CFU g^−1^ (BW-L), recommended 3.6 × 10^4^ CFU g^−1^ (B-BW), and high 7.2 × 10^4^ CFU g^−1^ (B-BW-H) concentrations of *B. subtilis*.

Parameters	BD ^a^	B-BW-L ^b^	B-BW ^a^	B-BW-H ^b^	PSE ^c^	*p*-Value
Alkaline phosphatase (U/L)	4.15	4.13	4.18	4.35	0.088	0.383
Alanine transaminase (U/L)	2.88	2.77	2.94	2.78	0.135	0.773
Gamma-glutamyl Transferase (U/L)	2.25	2.00	2.50	3.00	0.224	0.071
Bile acids (µmol/L)	23.50	24.33	12.75	19.33	4.313	0.247
Total Bilirubin (mg/dL)	0.75	0.73	0.83	0.80	0.026	0.104
Albumin (g/dL)	1.93	1.77	2.00	2.10	0.109	0.285
Blood urea nitrogen (mg/dL)	2.75	2.67	2.75	3.00	0.249	0.830
Cholesterol (mg/dL)	275.75	269.00	299.00	284.00	16.51	0.608
Hematocrit (%)	23.08	22.33	26.92	26.56	2.780	0.570

^a^*n* = 4, ^b^ *n* = 3, ^c^ PSE = Pooled Standard Error.

## Data Availability

The data that support the findings of this study are available from the corresponding author upon reasonable request.

## References

[1] Hargreaves J.A. (2002). Channel catfish farming in ponds: Lessons from a maturing industry. Rev. Fish Sci..

[2] (2021). United States Department of Agriculture: Stuttgart, AR, USA. National Agricultural Statistics Service Catfish Production 02/11/2022. https://usda.library.cornell.edu/concern/publications/bg257f046?locale=en.

[3] Pridgeon J., Klesius P. (2011). Molecular identification and virulence of three *Aeromonas hydrophila* isolates cultured from infected channel catfish during a disease outbreak in west Alabama (USA) in 2009. Dis. Aquat. Org..

[4] Mohammed H.H., Peatman E. (2018). Winter kill in intensively stocked channel catfish (*Ictalurus punctatus*): Coinfection with *Aeromonas veronii*, *Streptococcus parauberis* and *Shewanella putrefaciens*. J. Fish Dis..

[5] Shoemaker C.A., Olivares-Fuster O., Arias C.R., Klesius P.H. (2008). *Flavobacterium columnare* genomovar influences mortality in channel catfish (*Ictalurus punctatus*). Vet. Microbiol..

[6] Bilodeau A.L., Waldbieser G.C. (2005). Activation of TLR3 and TLR5 in channel catfish exposed to virulent *Edwardsiella ictaluri*. Dev. Comp. Immunol..

[7] Wagner B.A., Wise D.J., Khoo L.H., Terhune J.S. (2002). The epidemiology of bacterial diseases in food-size channel catfish. J. Aquat. Anim. Health.

[8] Chen H., Liu S., Xu X.R., Diao Z.H., Sun K.F., Hao Q.W., Liu S.S., Ying G.G. (2018). Tissue distribution, bioaccumulation characteristics and health risk of antibiotics in cultured fish from a typical aquaculture area. J. Hazard. Mater..

[9] Chen H., Liu S., Xu X.R., Liu S.S., Zhou G.J., Sun K.F., Zhao J.L., Ying G.G. (2015). Antibiotics in typical marine aquaculture farms surrounding Hailing Island, South China: Occurrence, bioaccumulation and human dietary exposure. Mar. Pollut. Bull..

[10] Watts J.E.M., Schreier H.J., Lanska L., Hale M.S. (2017). The rising tide of antimicrobial resistance in aquaculture: Sources, sinks and solutions. Mar. Drugs.

[11] Santos L., Ramos F. (2018). Antimicrobial resistance in aquaculture: Current knowledge and alternatives to tackle the problem. Int. J. Antimicrob. Agents.

[12] Citarasu T. (2009). Herbal biomedicines: A new opportunity for aquaculture industry. Aquacult. Int..

[13] Ringø E., Van Doan H., Lee S.H., Soltani M., Hoseinifar S.H., Harikrishnan R., Song S.K. (2020). Probiotics, lactic acid bacteria and bacilli: Interesting supplementation for aquaculture. J. Appl. Microbiol..

[14] Reuter K., Steinbach A., Helms V. (2016). Interfering with bacterial quorum sensing. Perspect. Medicin. Chem..

[15] Hai N.V. (2015). The use of probiotics in aquaculture. J. Appl. Microbiol..

[16] Harikrishnan R., Balasundaram C., Heo M.-S. (2011). Impact of plant products on innate and adaptive immune system of cultured finfish and shellfish. Aquaculture.

[17] Kumar G., Byars T.S., Greenway T.E., Aarattuthodiyil S., Khoo L.H., Griffin M.J., Wise D.J. (2019). Economic assessment of commercial-scale *Edwardsiella ictaluri* vaccine trials in U.S. catfish industry. Aquac. Econ. Manag..

[18] Pridgeon J.W., Klesius P.H. (2011). Development of a novobiocin-resistant *Edwardsiella ictaluri* as a novel vaccine in channel catfish (*Ictalurus punctatus*). Vaccine.

[19] Van Hai N. (2015). The use of medicinal plants as immunostimulants in aquaculture: A review. Aquaculture.

[20] Edwards P. (2015). Aquaculture environment interactions: Past, present and likely future trends. Aquaculture.

[21] Li Y., Boyd C.E. (2016). Influence of a bacterial amendment on water quality in small research ponds for channel catfish, *Ictalurus punctatus*, production. J. World Aquacult. Soc..

[22] Shelby R.A., Lim C., Yildirim-Aksoy M., Klesius P.H. (2007). Effects of probiotic bacteria as dietary supplements on growth and disease resistance in young channel catfish, *Ictalurus punctatus* (Rafinesque). J. Appl. Aquac..

[23] Luo Z., Bai X., Chen C. (2014). Integrated application of two different screening strategies to select potential probiotics from the gut of channel catfish *Ictalurus punctatus*. Fish Sci..

[24] Ringø E., Song S.K. (2016). Application of dietary supplements (synbiotics and probiotics in combination with plant products and β-glucans) in aquaculture. Aquacult. Nutr..

[25] Kumar V., Sinha A.K., Makkar H.P., De Boeck G., Becker K. (2012). Phytate and phytase in fish nutrition. J. Anim. Physiol. Anim. Nutr..

[26] Gatesoupe F.J. (1999). The use of probiotics in aquaculture. Aquaculture.

[27] Ran C., Carrias A., Williams M.A., Capps N., Dan B.C., Newton J.C., Kloepper J.W., Ooi E.L., Browdy C.L., Terhune J.S. (2012). Identification of *Bacillus* strains for biological control of catfish pathogens. PLoS ONE.

[28] Kloepper J.W., Ryu C.-M., Zhang S. (2004). Induced systemic resistance and promotion of plant growth by *Bacillus* spp. Phytopathology.

[29] Ibrahim F., Ouwehand A.C., Salminen S.J. (2004). Effect of temperature on *in vitro* adhesion of potential fish probiotics. Microb. Ecol. Health Dis..

[30] Thurlow C.M., Williams M.A., Carrias A., Ran C., Newman M., Tweedie J., Allison E., Jescovitch L.N., Wilson A.E., Terhune J.S. (2019). *Bacillus velezensis* AP193 exerts probiotic effects in channel catfish (*Ictalurus punctatus*) and reduces aquaculture pond eutrophication. Aquaculture.

[31] Srisapoome P., Areechon N. (2017). Efficacy of viable *Bacillus pumilus* isolated from farmed fish on immune responses and increased disease resistance in nile tilapia (*Oreochromis niloticus*): Laboratory and on-farm trials. Fish Shellfish Immunol..

[32] Boyd C.E., Romaire R.P., Johnston E. (1979). Water quality in channel catfish production ponds. J. Environ. Qual..

[33] Schmittgen T.D., Livak K.J. (2008). Analyzing real-time PCR data by the comparative C(T) method. Nat. Protoc..

[34] Moreira G., Shoemaker C., Zhang D., Xu D.H. (2017). Expression of immune genes in skin of channel catfish immunized with live theronts of *Ichthyophthirius multifiliis*. Parasite Immunol..

[35] Wang J., Xiong G., Bai C., Liao T. (2021). Anesthetic efficacy of two plant phenolics and the physiological response of juvenile *Ictalurus punctatus* to simulated transport. Aquaculture.

[36] Kordon A.O., Abdelhamed H., Ahmed H., Baumgartner W., Karsi A., Pinchuk L.M. (2019). Assessment of the live attenuated and wild-type *Edwardsiella ictaluri*-induced immune gene expression and Langerhans-like cell profiles in the immune-related organs of catfish. Front. Immunol..

[37] Jiang C., Zhang J., Yao J., Liu S., Li Y., Song L., Li C., Wang X., Liu Z. (2015). Complement regulatory protein genes in channel catfish and their involvement in disease defense response. Dev. Comp. Immunol..

[38] Jiang H., Wang M., Fu L., Zhong L., Liu G., Zheng Y., Chen X., Bian W. (2020). Liver transcriptome analysis and cortisol immune-response modulation in lipopolysaccharide-stimulated in channel catfish (*Ictalurus punctatus*). Fish Shellfish Immunol..

[39] Hao K., Yuan S., Yu F., Chen X.H., Bian W.J., Feng Y.H., Zhao Z. (2021). Acyclovir inhibits channel catfish virus replication and protects channel catfish ovary cells from apoptosis. Virus Res..

[40] Manera M., Britti D. (2006). Assessment of blood chemistry normal ranges in rainbow trout. J. Fish Biol..

[41] Bruce T.J., Ma J., Sudheesh P.S., Cain K.D. (2021). Quantification and comparison of gene expression associated with iron regulation and metabolism in a virulent and attenuated strain of *Flavobacterium psychrophilum*. J. Fish Dis..

[42] Banerjee G., Ray A.K. (2017). The advancement of probiotics research and its application in fish farming industries. Res. Vet. Sci..

[43] Jahangiri L., Esteban M.Á. (2018). Administration of probiotics in the water in finfish aquaculture systems: A Review. Fishes.

[44] Addo S., Carrias A.A., Williams M.A., Liles M.R., Terhune J.S., Davis D.A. (2017). Effects of *Bacillus subtilis* strains on growth, immune parameters, and *Streptococcus iniae* susceptibility in Nile tilapia, *Oreochromis niloticus*. J. World Aquacult. Soc..

[45] Addo S., Carrias A.A., Williams M.A., Liles M.R., Terhune J.S., Davis D.A. (2017). Effects of *Bacillus subtilis* strains and the prebiotic Previda® on growth, immune parameters and susceptibility to *Aeromonas hydrophilainfection* in nile tilapia, *Oreochromis niloticus*. Aquacult. Res..

[46] Peterson B., Wood M., Booth N., Morgan N., Tellez G., Hargis B. Feeding *Lactobacillus* spp. and *Bacillus* spp. Does not improve growth or survival of channel catfish experimentally challenged with *Edwardsiella ictaluri*. Proceedings of the American Society of Animal Science Annual Meeting.

[47] Merrifield D.L., Dimitroglou A., Bradley G., Baker R.T.M., Davies S.J. (2010). Probiotic applications for rainbow trout (*Oncorhynchus mykiss* Walbaum) I. Effects on growth performance, feed utilization, intestinal microbiota and related health criteria. Aquacult. Nutr..

[48] Hai N.V., Buller N., Fotedar R. (2009). Effects of probiotics (*Pseudomonas synxantha* and *Pseudomonas aeruginosa*) on the growth, survival and immune parameters of juvenile western king prawns (*Penaeus latisulcatus* Kishinouye, 1896). Aquacult. Res..

[49] Barton B.A. (2002). Stress in Fishes: A diversity of responses with particular reference to changes in circulating corticosteroids1. Integr. Comp. Biol..

[50] Faggio C., Piccione G., Marafioti S., Arfuso F., Trischitta F., Fortino G., Fazio F. (2014). Monthly variations of haematological parameters of *Sparus aurata* and *Dicentrarchus labrax* reared in Mediterranean land off-shore tanks. Cah. Biol. Mar..

[51] Panigrahi A., Kiron V., Satoh S., Watanabe T. (2010). Probiotic bacteria *Lactobacillus rhamnosus* influences the blood profile in rainbow trout *Oncorhynchus mykiss* (Walbaum). Fish Physiol. Biochem..

[52] Welker T.L., Lim C., Yildirim-Aksoy M., Shelby R., Klesius P.H. (2007). Immune response and resistance to stress and *Edwardsiella ictaluri* challenge in channel catfish, *Ictalurus punctatus*, fed diets containing commercial whole-cell yeast or yeast subcomponents. J. World Aquacult. Soc..

[53] Adorian T.J., Jamali H., Farsani H.G., Darvishi P., Hasanpour S., Bagheri T., Roozbehfar R. (2019). Effects of probiotic bacteria *Bacillus* on growth performance, digestive enzyme activity, and hematological parameters of asian sea bass, *Lates calcarifer* (Bloch). Probiot. Antimicrob. Proteins.

[54] Aly S.M., Abdel-Galil Ahmed Y., Abdel-Aziz Ghareeb A., Mohamed M.F. (2008). Studies on *Bacillus subtilis* and *Lactobacillus acidophilus*, as potential probiotics, on the immune response and resistance of tilapia nilotica (*Oreochromis niloticus*) to challenge infections. Fish Shellfish Immunol..

[55] Reda R.M., Selim K.M. (2015). Evaluation of *Bacillus amyloliquefaciens* on the growth performance, intestinal morphology, hematology and body composition of Nile tilapia, *Oreochromis niloticus*. Aquacul. Int..

[56] Chen Z., Ceballos-Francisco D., Guardiola F.A., Esteban M.Á. (2020). Dietary administration of the probiotic *Shewanella putrefaciens* to experimentally wounded gilthead seabream (*Sparus aurata* L.) facilitates the skin wound healing. Sci. Rep..

[57] Reyes-Cerpa S., Maisey K., Reyes-López F., Toro-Ascuy D., Sandino A.M., Imarai M. (2012). Fish cytokines and immune response. New Advances and Contributions to Fish Biology.

[58] Picchietti S., Fausto A.M., Randelli E., Carnevali O., Taddei A.R., Buonocore F., Scapigliati G., Abelli L. (2009). Early treatment with *Lactobacillus delbrueckii* strain induces an increase in intestinal T-cells and granulocytes and modulates immune-related genes of larval *Dicentrarchus labrax* (L.). Fish Shellfish Immunol..

[59] Grayfer L., Belosevic M. (2012). Cytokine regulation of teleost inflammatory responses. New Advances and Contributions to Fish Biology.

[60] Mohammadian T., Ghanei-Motlagh R., Molayemraftar T., Mesbah M., Zarea M., Mohtashamipour H., Jangaran Nejad A. (2021). Modulation of growth performance, gut microflora, non-specific immunity and gene expression of proinflammatory cytokines in shabout (*Tor grypus*) upon dietary prebiotic supplementation. Fish Shellfish Immunol..

[61] Zou J., Secombes C.J. (2016). The function of fish cytokines. Biology.

[62] Feng J., Li D., Liu L., Tang Y., Du R. (2019). Characterization and comparison of the adherence and immune modulation of two gut Lactobacillus strains isolated from *Paralichthys olivaceus*. Aquaculture.

[63] Hasan T., Jang W.J., Tak J.Y., Lee B.-J., Kim K.W., Hur S.W., Han H.-S., Kim B.-S., Min D.-H., Kim S.-K. (2018). Effects of *Lactococcuslactis* subsp. lactis I2 with β-glucooligosaccharides on growth, innate immunity and Streptococcosis resistance in olive flounder (*Paralichthys olivaceus*). J. Microbiol. Biotechnol..

[64] Köhidai L., Csaba G. (1998). Chemotaxis and chemotactic selection induced with cytokines (IL-8, Rantes and TNF-A) in the unicellular *Tetrahymena pyriformis*. Cytokine.

[65] Rodríguez I., Chamorro R., Novoa B., Figueras A. (2009). β-Glucan administration enhances disease resistance and some innate immune responses in zebrafish (*Danio rerio*). Fish Shellfish Immunol..

[66] Lu Z.-Y., Jiang W.-D., Wu P., Liu Y., Kuang S.-Y., Tang L., Yang J., Zhou X.-Q., Feng L. (2020). Mannan oligosaccharides supplementation enhanced head-kidney and spleen immune function in on-growing grass carp (*Ctenopharyngodon idella*). Fish Shellfish Immunol..

[67] Kawai T., Akira S. (2006). TLR signaling. Cell Death Differ..

[68] Liu S., Wang S., Cai Y., Li E., Ren Z., Wu Y., Guo W., Sun Y., Zhou Y. (2020). Beneficial effects of a host gut-derived probiotic, *Bacillus pumilus*, on the growth, non-specific immune response and disease resistance of juvenile golden pompano, *Trachinotus ovatus*. Aquaculture.

[69] Wang Q., Shen J., Yan Z., Xiang X., Mu R., Zhu P., Yao Y., Zhu F., Chen K., Chi S. (2020). Dietary *Glycyrrhiza uralensis* extracts supplementation elevated growth performance, immune responses and disease resistance against *Flavobacterium columnare* in yellow catfish (*Pelteobagrus fulvidraco*). Fish Shellfish Immunol..

